# Immediate bleeding complications in dental implants: A systematic review

**DOI:** 10.4317/medoral.20203

**Published:** 2014-12-05

**Authors:** José-Carlos Balaguer-Martí, David Peñarrocha-Oltra, José Balaguer-Martínez, Miguel Peñarrocha-Diago

**Affiliations:** 1 Degree in Dental Surgery. Resident of the Master in Oral Surgery and Implantology. Department of Stomatology. Medical and Dental School, Universitat of València; 2 Master in Oral Surgery and Implantology. Department of Stomatology. Valencia University Medical and Dental School, Universitat of València; 3 Associate Professor of Oral Surgery. Department of Stomatology. Medical and Dental School, Universitat of València; 4 Chairman of Oral Surgery. Department of Stomatology. Valencia University Medical and Dental School, Universitat of València

## Abstract

Objective: A review is made of the immediate or immediate postoperative bleeding complications in dental implants, with a view to identifying the areas of greatest bleeding risk, the causes of bleeding, the length of the implants associated with bleeding, the most frequently implicated blood vessels, and the treatments used to resolve these complications. Material and Methods: A Medline (PubMed) and Embase search was made of articles on immediate bleeding complications in dental implants published in English up until May 2014. Inclusion criteria: studies in humans subjects with severe bleeding immediately secondary to implant placement, which reported the time until the hemorrhage, the implant lenght, the possible cause of bleeding and the treatment. Exclusion criteria: patients receiving anticoagulation treatment. Results: Fifteen articles met the inclusion criteria. The area with the largest number of bleeding complications corresponded to the mandibular canine. The cause of bleeding was lingual cortical bone perforation during implant placement, with damage to the sublingual artery. The implants associated with bleeding were those measuring 15 mm in length or more. Management focused on securing the airway (with intubation or tracheostomy if necessary), with bleeding control. Conclusions: It’s important to pay special attention when the implants are placed in the mandibular anterior zone, especially if long implants are used. The most frequently cause of bleeding was the perforation of the lingual plate. Treatment involves securing the airway, with bleeding control.

** Key words:**Hemorrhage, complications, immediate, bleeding, dental implants.

## Introduction

Complications in dental implant surgery are infrequent and normally self-limiting, causing the technique to become an almost routine procedure. However, immediate bleeding complications have been described which although infrequent, may prove serious, particularly in the floor of the mouth. Some cases can even be life-threatening, requiring emergency treatment on an in-hospital basis (1-3).

The branches of the sublingual and sub mental arteries that irrigate the lingual zone of the mandible are located close to the lingual cortical plate. This implies an increased risk of bleeding if the lingual cortical bone is damaged during drilling or implant placement. Profuse bleeding is less common in the upper jaw, and only one article to date has reported important bleeding following maxillary sinus lift surgery with immediate implant placement (4).

Adequate planning of surgery is required, with thorough knowledge of the anatomical features of the surgical zone, and the use of complementary techniques such as cone-beam computed tomography (CBCT), in order to avoid possible risk situations. Despite such precautions, however, some patients are at an increased risk of bleeding due to physiological anatomical variants (5). Two reviews have been published on bleeding complications associated to implant placement: one in the anterior mandibular zone (6) and the other in the general region of the mouth (7). These studies indicate that despite its apparent simplicity, implant placement is not without risks, which in some cases may prove serious. Since the publication of these reviews, three new articles on bleeding complications after dental implant placement have appeared in the literature: two in the mandibular region (3,8) and one in the upper jaw (4).

The present study reviews the literature on immediate or immediate postoperative bleeding complications in dental implants, with a view to identifying the areas of greatest bleeding risk, the causes of bleeding (including the implicated arteries), the length of the implants associated with bleeding, and the treatments provided.

## Material and Methods

- Study search strategies

The review was based on the following question: Is implant length and location associated to important bleeding risk during or immediately after implant placement? A Medline (PubMed) and Embase search was made of articles on immediate bleeding complications in dental implants published in English up until May 2014. The following key words were used: “(hemorrhag* OR bleeding) AND complications) AND dental implants”, “hemorrhag* AND complications AND pterygoid”. The search was completed with a review of the references cited by the identified articles, in order to generate additional studies not identified by the online search. Important bleeding complications were defined as bleeding requiring hospital admission. The reviewers (JB and DP) individually analyzed the titles of the articles, with application of the inclusion and exclusion criteria (defined below) in order to identify the publications to be included in the systematic review. The reviewers then performed a new selection of articles after analyzing the abstracts of those manuscripts that had been selected according to title. Lastly, a full-text review was made. Article inclusion was decided by consensus in the event of disagreement between the reviewers. The Mendeley Desktop reference manager (Mendeley Ltd., London, UK) was used.

- Inclusion and exclusion criteria

The following search inclusion criteria were used: (1) articles published in English; (2) studies in humans; (3) availability of the full-length article; (4) specification of the time from implant placement to bleeding; and (5) specification of the treatment used to solve the complication. As exclusion criterion, we excluded studies involving patients receiving anticoagulation treatment.

After analyzing the articles, the reviewers collected the following information from each publication: Number of patients, Age, Artery causing bleeding, Position of the implant causing bleeding, Time from implant bed drilling to bleeding, Length of the implant and treatment provided.

## Results

**Figure 1:**
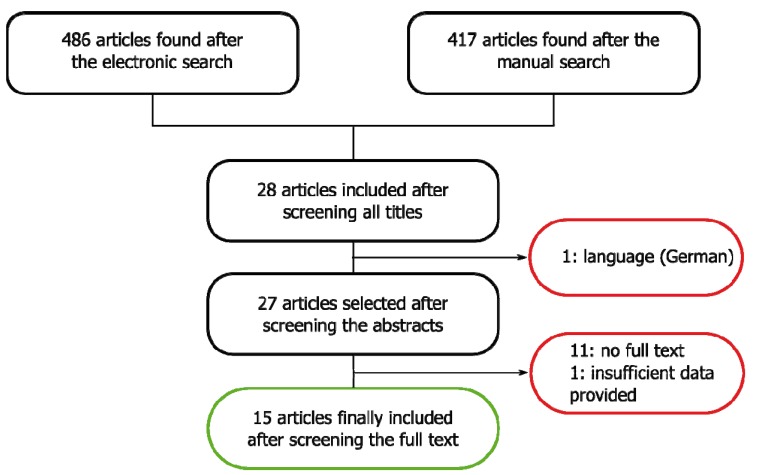
Article screening flowchart.

A total of 486 articles were identified. After reviewing the abstracts and eliminating duplicities, this number was reduced to 28 publications, of which 13 were excluded because they failed to meet the inclusion criteria: one article was published in German, 11 could not be retrieved as the full-length article, and one failed to produce all the information required for inclusion (Fig. 1). A total of 15 articles were thus finally included in the review ( Table 1). The mean patient age was 60 years. The gender distribution was specified in 9 articles (1-4,8-12) (55.5% females and 44.5% males).

**Table 1:**
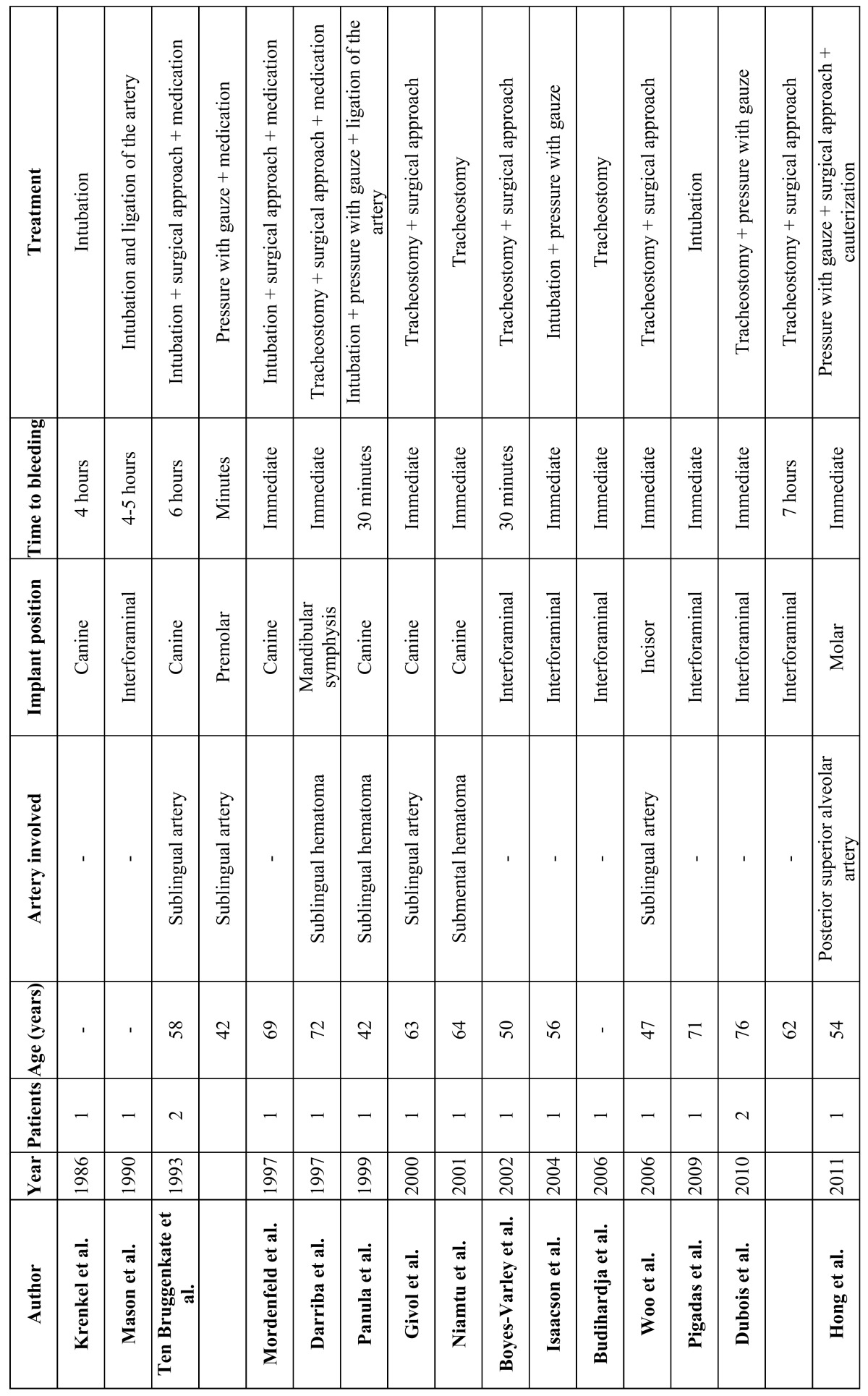
Publications included in the systematic review. Arteries possibly involved in bleeding, position of the implants and treatment.

Immediate bleeding complications were most often observed in the mandibular canine region (6 cases) (9-11,13-15), followed by the incisor zone (2 cases) (1,2) and the premolars (1 case) (13). In one-half of the cases the precise location of the implant causing bleeding was not indicated.

The possible cause of bleeding was only specified in 7 of the case series included in the review (1,2,4,10,11,13,14). The most common cause was perforation of the lingual plate, causing damage to the sublingual artery. In those cases in which the length of the implant causing bleeding was specified, the length was always 15 mm or longer (6,9,10,12,16,17).

Gauze was applied to the bleeding zone under manual pressure (3,6,11,13,14,16), though this maneuver only proved effective in one case (13). Securing the airway was the priority concern of treatment. Forty-one percent of the patients were intubated (8,9,12-16), and a tracheotomy proved necessary in 47% of the cases (1-3,10,11,17,18).

After securing the airway, Drainage of the hematoma was also carried out (1-3,9,10,12,13,15-18), as well as ligation of the bleeding artery (1-3,9,10,12,16,18). In some cases ligation of the facial and lingual arteries proved necessary to control the bleeding, due to the existence of anastomoses between the vessels - particularly in the mandibular symphyseal region (7). Some authors prescribed oral or intravenous antibiotics (1,8,9,12,13). None of the reviewed articles reported fatalities.

We identified only one case of serious bleeding of the upper jaw in the context of immediate dental implant placement following indirect sinus lift surgery. Treatment consisted of the application of gauze to the bleeding zone, but this was not enough to stop the bleeding. A window was opened in the lateral wall of the maxillary sinus using the Caldwell-Luc technique, with cauterization of the posterior superior alveolar artery (4). None of the reviewed articles reported heavy bleeding immediately after implant placement in the pterygoid region.

## Discussion

All of the reviewed studies involved a single patient, except the articles published by Ten Bruggenkate *et al*. (13) and Dubois *et al*. (3), which included two patients each. This may be because these bleeding complications are very infrequent. However, compared with the large number of implants placed worldwide, the few reported cases of serious bleeding complications may be indicate of an underreporting of cases.

- Immediate bleeding complications in the mandible 

- Location

The dental implants causing important bleeding complications were most often located in the mandible in the canine region, followed by the incisors and first premolar zone. Many authors have studied the location of the most important blood vessels in the mandible, and their relation with the mandibular alveolar nerve and the cortical plate ( Table 2). Such bleeding is explained by the presence of the sublingual and sub mental arteries in these areas. In the study published by Mardinger *et al*. (19), the median distance from the sublingual and sub mental arteries to the alveolar crest was found to be 15 mm in the region of the incisors and canines. These authors also studied the distance from the mentioned arteries to the lingual cortical plate, which was found to be 4 mm in the chin region and 2 mm in the area of the canines and molars. Taking these observations into account, the canine region is identified as the most vulnerable zone, because it is here where the arteries run closest to the lingual plate and alveolar crest.

**Table 2:**
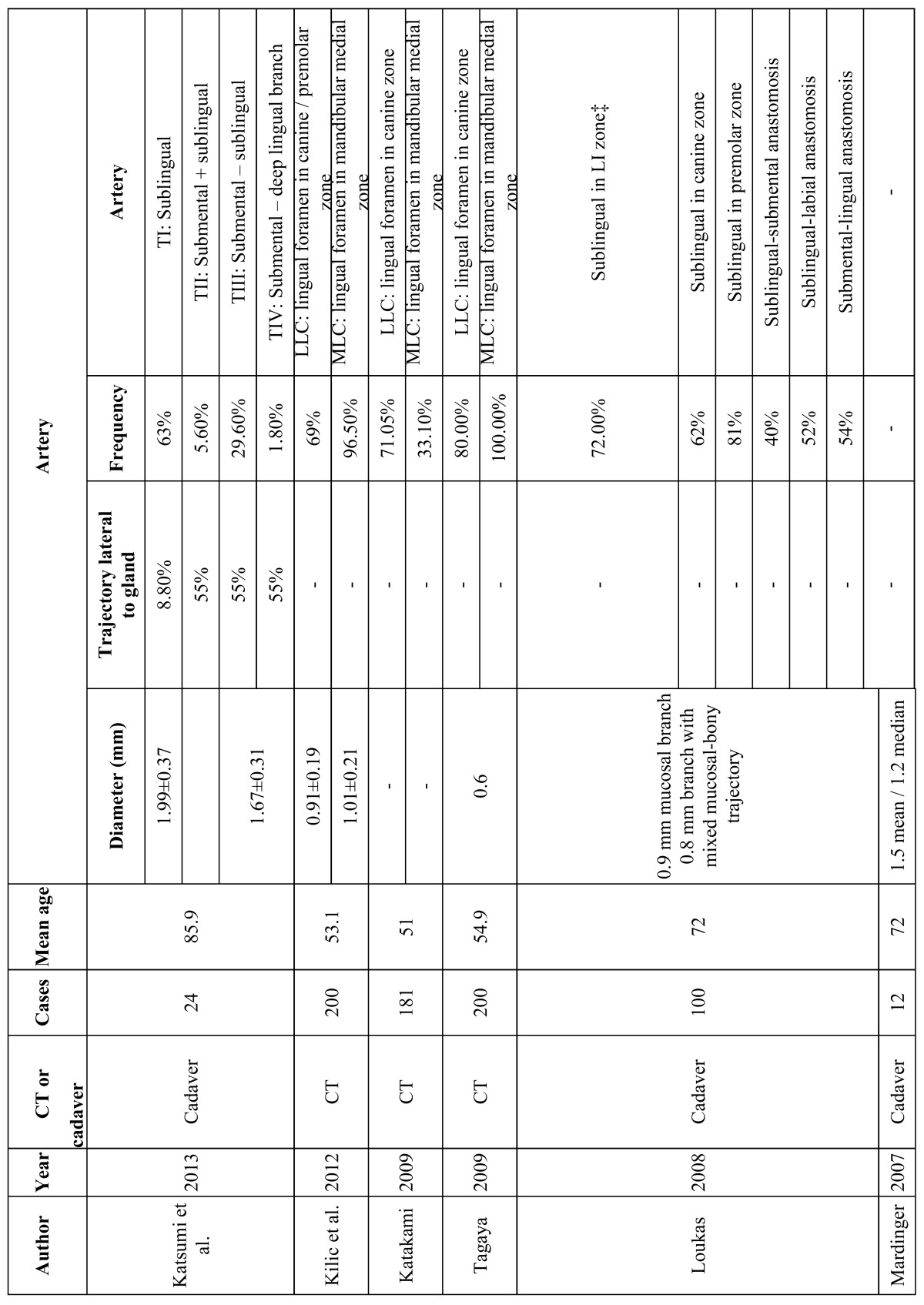
Location of the arteries possibly implicated in bleeding complications in the mandibular region, their trajectory and diameter according to radiographic and anatomical studies. *: Lateral lingual canal (LLC). †: Medial lingual canal (MLC). ‡: Lateral incisor (LI).

Anatomical and radiographic studies (20-22) identified lingual vascular canals in the mandible. Lateral lingual canals (LLCs) were present in the area of the canines in 69% (20), 71% (21) and 80% (22) of the cases, and the frequency of medial lingual canals (MLCs) varied between 33.1% and 96.5-100% (20,22) in the region of the mandibular incisors. The location of the lingual canals coincided with the most frequent sites of important bleeding during implant placement – the mandibular incisor and canine areas being the zones of greatest risk. The diameter of the canals was about 1.2 mm, which is enough to produce severe sublingual bleeding (23). Katakami *et al*. (21) described the presence of anastomoses between the lingual canals and the inferior alveolar nerve in 20.1% of the cases. This may imply increased risk, due to the greater difficulty of controlling the bleeding.

- Implant length.

All the implants causing bleeding were 15 mm in length or longer in those articles in which implant length was specified. Mardinger *et al*. (19) found the median distance from the mandibular blood vessels to the alveolar crest to be 15 mm in the mandibular incisor and canine regions, and 19 mm in the zone of the molars. It is therefore advisable to use shorter implants in the incisor and canine regions, due to the greater proximity of the arteries to the alveolar crest. Most of the blood vessels above the mylohyoid muscle were located in the canine zone (68.7%) – thus increasing the risk of bleeding.

- Time to bleeding.

Bleeding can occur immediately upon implant placement (70.6% of the patients) (1-4,8-12,18) or in the immediate postoperative period and up to 7 hours after surgery (3,13,15,16). A possible explanation for this is that a lacerated artery bleeds slowly but persistently, while full arterial sectioning can give rise to vasospasm which, combined with the use of vasoconstrictors during surgery, can delay bleeding for several hours (23).

- Cause of bleeding.

In the reviewed case series, most of the authors pointed to sublingual artery damage secondary to perforation of the lingual cortical plate as the cause of bleeding (1,2,10,13,14). Katsumi *et al*. (24), in an anatomical study, classified the anatomical variants of the sublingual and submental branches and their trajectories into four types. In type I, the most common presentation, the artery typically runs medial to the sublingual gland (92% of the cases), in contrast to types II-IV (45% of the cases), and lies further from the lingual plate than in the other types. This means that the risk of arterial damage is greater in the presence of types II-IV, as a result of increased proximity to the lingual plate. In the case of type I the risk is smaller, since the artery is located further from the lingual plate and runs medial to the sublingual gland. The diameter of these vessels ranged from 1.7-2 mm and was not related to patient age (24).

- Immediate bleeding complications in the upper jaw.

We found only one article describing serious bleeding in the upper jaw after implant placement. The cause was considered to be damage to the posterior superior alveolar artery. Radiographic and anatomical studies have defined the position of the arteries in the region of the maxillary sinus, and the anastomoses among them (5,25-29). Anastomoses were found between the alveolar antral artery (AAA) - a branch of the posterior superior alveolar artery – and the infra orbital artery (IOA) in 100% of the anatomical studies in cadavers. However, such anastomoses were only found in 44-55% of the examined computed tomography scans ( Table 3). The proposed explanation for this discrepancy between the anatomical study and the computed tomography findings, despite the fact that the same cadavers were involved, was that the trajectory of the anastomosis is not always strictly intraosseous. In all the anatomical studies the trajectory was partially intraosseous, and it proved difficult to establish by computed tomography whether the anastomosis ran through the Schneiderian membrane instead of maxillary bone (26). Other authors have reported similar findings (29). The mean diameter of the anastomoses between the AAA and IAO was 1 mm (5,25,26,28), and did not decrease with age (29). The distance from the anastomosis to the alveolar crest ranged from 11,2 mm to 19 mm (5,25,26,28). Solar *et al*. (29) determined the mean distance from the anastomosis to the alveolar crest over both its intraosseous and extraosseous trajectories independently – recording values of 19 mm and 24.5 mm, respectively. Elian *et al*. (5) estimated that 20% of the osteotomies performed in the usual position in sinus lift surgery have an increased risk of bleeding complications. Lamas *et al*. (7) recommended the use of osteotomes instead of drilling, where possible, if implants are to be placed in risk areas of the upper jaw.

**Table 3:**
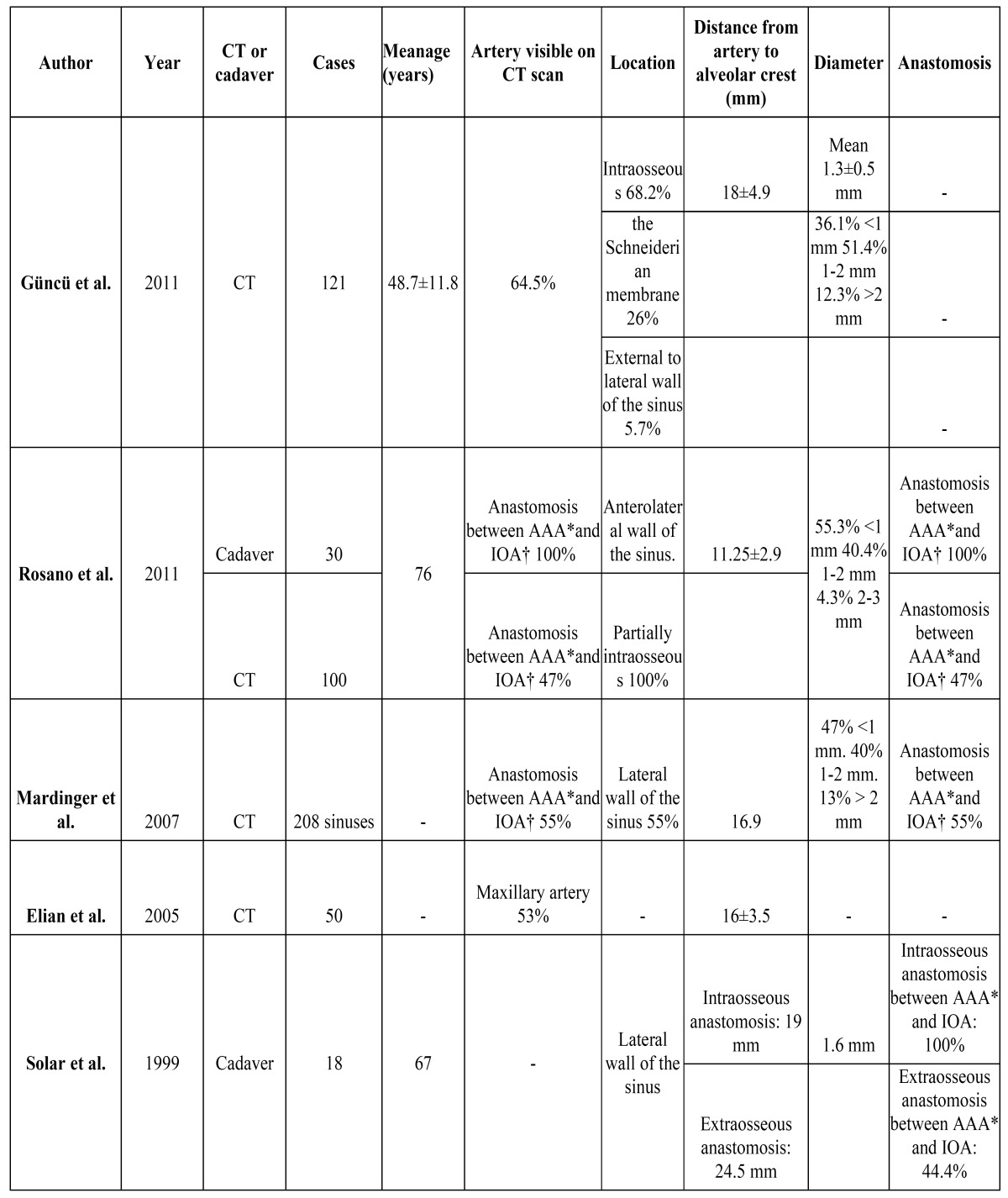
Anatomical and radiographic studies of the maxillary blood vessels most often involved in severe bleeding complications. Frequency, diameter of the arteries, distance to the alveolar crest and anastomosis.*: Alveolar antral artery (AAA). †: Infraorbital artery (IOA).

## Conclusions

Bleeding complications after dental implant placement are infrequent but can be serious, particularly in the anterior mandibular region. The most common cause of heavy bleeding in the mandibular zone is lingual cortical bone perforation, with damage to the sublingual artery - in all cases on placing long implants (15 mm or more in length). Treatment involves securing the airway, with bleeding control. The use of short implants is advised in the anterior mandibular sector, in order to avoid the risk of important bleeding complications.
